# Cohort design transparency is crucial for TriNetX-based studies

**DOI:** 10.1016/j.jvscit.2026.102189

**Published:** 2026-02-24

**Authors:** Joshua Wang

**Affiliations:** Department of Research, Taipei Tzu Chi Hospital, Buddhist Tzu Chi Medical Foundation, New Taipei City, Taiwan; School of Biomedical Sciences, Queensland University of Technology, Brisbane, Australia

I enjoyed reading Talukder et al’s^1^ retrospective comparison of outcomes after either endovascular or open surgical repair of the descending thoracic aorta using the TriNetX network. I am concerned that the authors have not provided enough methodological details to understand exactly how their patient cohorts were constructed. The authors provide the clinical codes used to define their outcomes, and state that the codes used to construct their cohorts are available in their attached Appendix. However, this file does not define any of the clinical codes used. Furthermore, the temporal relationship between the inclusion and exclusion criteria for each cohort is not well-defined.

These details can greatly influence the findings of TriNetX-based studies.^2^ For example, in the comparison of repair approaches for unruptured aneurysms, it is unclear if the exclusion of aneurysm rupture is set to before the index surgery or for the entirety of the patient's record. If the latter was performed, then this may underestimate the rate of complications because it would exclude patients with subsequent ruptures resulting from surgical complications such as endoleak.^3^

When an outcome analysis is run on TriNetX, the user can export a word doc summary of the study, which includes tables of the exact cohort designs used in the analysis (Table). I would encourage the authors of the study to provide these tables, allowing for readers to more deeply engage with their findings.

**Table:** Example exported cohort design demonstrating endovascular repair patients without a prior rupture

Group 1				
Group 1A				
Must have	Any of	Procedure	UMLS:CPT:1006313	Endovascular repair procedures of the descending thoracic aorta
		Procedure	UMLS:CPT:1006314	Endovascular repair of descending thoracic aorta (eg, aneurysm, pseudoaneurysm, dissection, penetrating ulcer, intramural hematoma, or traumatic disruption)
Cannot have		Procedure	UMLS:ICD10PCS:02QW0ZZ	Repair thoracic aorta, descending, open approach
	or	Diagnosis	UMLS:ICD10CM:I71.012	Dissection of descending thoracic aorta
Date constraint		The terms in this group occurred between January 1, 2000, and June 14, 2025		
Event relationship		Any instance of group 1B occurred on or before the first instance of group 1A		
Group 1B				
Cannot have		Diagnosis	UMLS:ICD10CM:I71.13	Aneurysm of the descending thoracic aorta, ruptured
	or	Diagnosis	UMLS:ICD10CM:I71.8	Aortic aneurysm of unspecified site, ruptured
	or	Diagnosis	UMLS:ICD10CM:I71.51	Supraceliac aneurysm of the thoracoabdominal aorta, ruptured
	or	Diagnosis	UMLS:ICD10CM:I71.52	Paravisceral aneurysm of the thoracoabdominal aorta, ruptured
	or	Diagnosis	UMLS:ICD10CM:I71.11	Aneurysm of the ascending aorta, ruptured

This design includes both the clinical codes used as inclusion/exclusion criterion, and the temporal relationships between those codes.

## Funding

None.

## Disclosures

None.
